# Long-Acting Injectable Antipsychotics in Adolescents: From Current Evidence and Gaps to Clinical Practice

**DOI:** 10.3390/ph18101571

**Published:** 2025-10-18

**Authors:** Simone Pardossi, Alessandro Cuomo, Giacomo Gualtieri, Mario Pinzi, Andrea Fagiolini

**Affiliations:** 1 Department of Molecular Medicine, University of Siena School of Medicine, 53100 Siena, Italy; alessandro.cuomo@unisi.it (A.C.); mario.pinzi@student.unisi.it (M.P.); andrea.fagiolini@unisi.it (A.F.); 2 Department of Medicine, Surgery and Neuroscience, University of Siena, 53100 Siena, Italy; giacomo.gualtieri2@unisi.it

**Keywords:** adolescents, long-acting injectable antipsychotics, schizophrenia, bipolar disorder, nonadherence, safety

## Abstract

**Background:** Adolescence is a vulnerable period for the onset of severe psychiatric conditions, such as psychotic spectrum disorders. Non-adherence to antipsychotics is a common problem in young people with these conditions and paves the way for relapse, rehospitalization, and functional impairment. Co-occurring substance use disorders (SUDs) further undermine adherence and worsen outcomes. Long-acting injectable antipsychotics (LAIs) improve adherence and outcomes in adults, but none are licensed for use in individuals under 18. This review seeks to distill the available evidence on LAIs’ use in adolescents, from efficacy to safety, and to outline clinical practice recommendations. **Methods:** A narrative review was conducted. The evidence was organized by drug class: risperidone, paliperidone, aripiprazole, and other antipsychotics (olanzapine, haloperidol, first-generation depots). **Results:** Evidence in adolescents remains sparse and heterogeneous. Risperidone LAI has shown improvements in symptom severity, functioning, and behavioral control in bipolar disorder and schizophrenia, though commonly associated with side effects. Paliperidone palmitate demonstrated benefit in first-episode schizophrenia and autism spectrum disorder with intellectual disability, reducing hospital use but carrying risks of EPS and hyperprolactinemia. Aripiprazole LAI showed functional gains, short-term tolerability, and encouraging acceptance in case reports. Other LAIs were used in highly resistant cases with some clinical benefit, though extrapyramidal adverse events were common. **Conclusions:** The current literature provides limited data, and no clinical guidelines exist for the use of LAI in adolescents. Nonetheless, off-label use is reported in selected cases in clinical practice. Best practice is to start with oral stabilization, then use the lowest effective LAI with psychosocial support and close monitoring. When SUD co-occurs, LAIs may also help mitigate risks related to misuse/diversion of oral medication, provided that care includes systematic SUD screening and early intervention. Prospective controlled studies are urgently needed to establish long-term efficacy and safety in this vulnerable population.

## 1. Introduction

### 1.1. Adolescents and Non-Adherence

The treatment of severe mental illnesses, including schizophrenia, schizoaffective disorder, and bipolar disorder, in adolescents faces the widespread problem of medication non-adherence [1]. The combination of early disease onset with active neurodevelopmental, psychological, and social changes in youth creates an environment in which substandard treatment produces more severe risks [2,3]. Moreover, non-adherence is an important factor that should not be undervalued, especially in adolescents with psychotic symptoms: patients who do not follow their prescribed oral antipsychotic medications experience elevated relapse rates, require more hospitalizations, and develop worse functional abilities, which in turn affect their academic, social, and occupational performance [4]. The main reasons for non-adherence in adolescents include poor understanding of their illness, the side effects of medication such as weight gain and sedation, cognitive problems, lack of family and social support, discrimination, and co-occurring substance use disorders [4,5,6]. The negative effects of non-adherence extend beyond clinical relapse, as it leads to faster functional deterioration, impaired neurocognitive growth, and higher healthcare expenses due to emergency care and hospital readmissions [4,5,6]. Moreover, adolescents who may require antipsychotic therapy, such as those with early-onset psychotic and bipolar disorders, frequently present with co-occurring substance use, most notably cannabis and alcohol [7,8]. These comorbidities are associated with earlier disease onset, greater symptom severity, and faster functional decline over time [7,8]. Affected patients often struggle to maintain regular treatment, and non-adherence substantially increases the risk of relapse and rehospitalization [9]. In first-episode psychosis cohorts, persistent cannabis use after illness onset has been shown to undermine medication adherence and predict recurrence of psychosis, highlighting substance use as a key modifiable driver of poor outcomes [9].

### 1.2. Long-Acting Injectable (LAI) Antipsychotics: Rationale, Pharmacokinetics, and Pharmacodynamics

Long-acting injectable (LAI) antipsychotics, depot formulations given periodically rather than daily, have already transformed the lives of many adults with schizophrenia by improving treatment adherence [10,11,12]. Yet, the benefits of LAIs extend beyond adherence alone, as their distinct pharmacokinetic properties also confer important clinical advantages [12]. Owing to their slow absorption and prolonged half-life, LAIs markedly reduce the peak-to-trough fluctuations in plasma drug concentrations typically observed with daily oral dosing. This results in steadier systemic exposure and tighter control within a narrower therapeutic range [12].

After intramuscular or subcutaneous injection, LAIs form a depot at the injection site, from which the drug is slowly and continuously absorbed into systemic circulation. Because this absorption process is typically slower than elimination, the absorption rate becomes the rate-limiting step in determining plasma concentrations—this phenomenon is known as flip-flop kinetics [12,13,14]. As a consequence, LAIs maintain more stable plasma levels, reduce peak-to-trough variability, and mitigate abrupt drops in drug concentration that might precipitate relapse, thereby enabling more consistent receptor engagement over time [12,13,14]. The “apparent” half-life of a depot formulation primarily reflects the prolonged absorption phase, rather than the drug’s true elimination half-life, as seen with oral dosing [14].

From a pharmacodynamic perspective, most antipsychotics—including LAIs—share a core mechanism: antagonism (or partial agonism) at dopamine D_2_ receptors, often combined with serotonin 5—HT_2_A receptor modulation, which helps balance efficacy on positive symptoms and tolerability for negative/cognitive symptoms [15]. Additional receptor affinities (e.g., α—adrenergic, histaminergic, muscarinic) vary across compounds and influence side—effect profiles [15]. For instance, risperidone and its active metabolite act primarily as D_2_ and 5—HT_2_A antagonists, with secondary activity on α_1_/α_2_—adrenergic receptors [16]. Pharmacokinetic modeling of risperidone LAI formulations has demonstrated how they can maintain therapeutic receptor occupancy with lower fluctuations compared to oral dosing [17].

The clinical relevance of these pharmacokinetic features is straightforward to grasp. Large peaks in plasma concentration can provoke transient but disabling side effects, such as sedation or EPS. Conversely, troughs may lower drug levels below therapeutic thresholds, leading to breakthrough symptoms or relapse. Over time, LAIs help maintain more even plasma concentrations, limiting both extremes. In this way, they not only secure adherence but also smooth tolerability profiles and may enhance overall acceptability [12].

Taken together, LAIs combine two essential advantages: they provide continuous therapeutic drug exposure while sparing patients the burden of disruptive peak effects [12].

In adults, extensive evidence shows that LAIs can shorten periods of untreated psychosis, prevent relapse, and even improve quality of life compared with oral formulations [18]. Given these advantages, it is natural to ask whether adolescents with early-onset psychotic or bipolar disorders might also benefit from LAI therapy—particularly those with pronounced non-adherence due to poor insight, cognitive immaturity, or reluctance to take daily medication [3].

Against this background, LAI offer practical benefits for adolescents with dual diagnoses: they reduce the burden of daily pill-taking, allow early detection of missed doses through treatment records, and are associated with lower risks of treatment interruption, relapse, and hospitalization than oral formulations [19]. Importantly, by limiting access to take-home pills, LAIs may also reduce the likelihood of intentional misuse, overdose, or diversion, a clinically relevant concern given reports of antipsychotic misuse and poisoning in adolescents [20].

Despite this rationale, no LAI is currently FDA-approved for patients under 18 [3]. No randomized controlled trials (RCTs) in this population have yet been conducted. Nevertheless, in recent years an increasing number of observational studies and small-scale trials have begun to document the off-label use of second-generation LAIs in adolescents. These newer studies, together with accumulated clinical experience, suggest that LAIs may be safe and effective in carefully selected youths. The clinical interest in LAIs is deeply rooted in their pharmacokinetic properties—such as prolonged half-life, reduced peak–trough variability, and predictable plasma levels—resulting from depot formulations. These features contribute to improved adherence and potentially lower relapse rates. However, their use in adolescents remains off-label, and the lack of pharmacovigilance data in this population reflects a broader regulatory gap in pediatric drug development. The aim of this narrative review is threefold: (1) to summarize existing evidence on the off-label use of LAI in adolescents; (2) to identify key research gaps in this area; and (3) to offer preliminary clinical recommendations based on the available literature and current clinical experience. This narrative review was conducted by searching PubMed, Scopus, and Web of Science databases up to September 2025. The following keywords and Boolean operators were used: (“long-acting injectable antipsychotics” OR “LAIs”) AND (“adolescents” OR “youth” OR “pediatric”) AND (“schizophrenia” OR “bipolar disorder” OR “psychosis”). Additional terms such as “nonadherence,” “safety,” and individual drug names (e.g., risperidone, paliperidone, aripiprazole) were used to refine the search. Eligible studies included peer-reviewed case reports, case series, observational studies, open-label trials, and retrospective cohorts that reported on LAI use in individuals under the age of 18.

## 2. Risperidone

In pediatric and adolescent populations, risperidone LAI has been explored almost exclusively in off-label contexts, often as a response to poor adherence, illness severity, or resistance to oral treatment (Table 1). A naturalistic six-month study in Italy included 19 patients aged 11–17 years with early-onset bipolar disorder, all prescribed 25–37.5 mg of risperidone LAI every two weeks [21]. Significant improvements were observed in both Clinical Global Impression Severity (CGI-S) and Children’s Global Assessment Scale (CGAS) scores, alongside marked reductions in manic, depressive, and psychotic symptoms. Importantly, no severe metabolic or laboratory changes were reported, although some patients presented increased appetite, weight gain, and high plasma levels of prolactin. No other severe adverse events occurred, supporting the drug’s efficacy and tolerability in this difficult-to-treat population [21].

Further evidence comes from Turkey, where risperidone LAI was evaluated in a retrospective series of 11 adolescents (mean age 14.9 years) [22]. Over 24 weeks, CGI-S scores fell from 6.55 to 2.18 (*p* < 0.001) and Clinical Global Impression Improvement (CGI-I) changed from 2.55 (week 8) to 1.91 (week 24; *p* = 0.001). Body weight increased modestly but not significantly (55.5 → 57.0 kg; *p* = 0.076). Extrapyramidal symptoms (EPS) were generally mild; the proportion with no EPS rose from 18.2% at week 8 to 54.5% at week 24. Overall, the study supports risperidone LAI as a feasible and well-tolerated option for adolescents with severe behavioral problems and poor adherence to oral therapy [22].

A smaller open-ward retrospective study [23] specifically examined adolescents (*n* = 14), mean age 13.9 years (SD: 2.9) with conduct disorder who had demonstrated pronounced non-adherence to oral medication. Comorbidities were common, including substance use disorders (SUD), Attention Deficit Hyperactivity Disorder (ADHD), and major depressive disorder (MDD). Most patients were started on risperidone LAI 25 mg every two weeks. During the hospitalization, significant reductions in aggression and disruptive behavior were observed, accompanied by corresponding improvements in overall functioning. Reported adverse effects included drowsiness, weight gain, and muscle stiffness, but no severe or unexpected events occurred. These findings suggest that long-acting formulations may also be useful in enhancing adherence and controlling severe behavioral symptoms in non-psychotic adolescents [23].

Additional observational data from a Spanish adolescent inpatient unit. 30 patients (mean age: 16.3, SD: 1.3) were treated with second-generation LAIs, including aripiprazole (40%), risperidone (36.7%), and paliperidone palmitate (23.3%). The majority (70%) had psychotic disorders, while 30% presented with disruptive behavior disorders, and psychiatric comorbidity was highly prevalent (93%), most frequently cannabis use disorder (53%). Over the course of hospitalization, CGAS scores improved significantly by a mean of 32 points (*p* < 0.001), with no differences between the three LAIs in terms of functional outcome. Adverse effects were reported by 23.3% of patients and were significantly more common with risperidone compared to aripiprazole (*p* = 0.014). Nevertheless, no patients discontinued treatment due to tolerability issues, and overall safety was deemed acceptable [24].

In a Chinese open-label 24-week trial, 31 adolescents (mean age 15.9 years, SD: 3.3) with early-onset schizophrenia, previously stabilized on oral risperidone or olanzapine, were switched to risperidone LAI every two weeks (25–50 mg). About 80% completed the study, with discontinuations mainly for side effects or insufficient response. By week 24, Positive and Negative Syndrome Scale (PANSS) and CGI-S scores improved significantly, and over two-thirds achieved at least a 20% reduction in PANSS. Adverse effects were mostly mild—such as depression, anxiety, headache, and insomnia—while EPS were rare. Prolactin decreased in those switched from risperidone but increased after olanzapine, and weight changes followed a similar pattern. No relevant ECG or laboratory abnormalities were reported. Overall, the study supports risperidone LAI as an effective and tolerable option for adolescent schizophrenia, with potential additional metabolic and endocrine benefits depending on prior treatment [25].

Early-onset schizophrenia patients treated with risperidone LAI at a hospital in Japan were compared with adults [26]. Twelve adolescents were included (mean age 16.8 years, SD: 1.7), and the average dose in this group was 37.5 mg every two weeks, somewhat lower than in adults. After one year, treatment retention was about 30% for adolescents compared with roughly 60% for adults. Although the difference was not statistically significant, there are reasons to believe that young people and adults face very different prospects when it comes to remaining on risperidone LAI. Among adolescents, discontinuation was attributed to patient decision (25%), adverse effects (25%), and lack of efficacy (17%), with adverse events accounting for around 40% of cases—a rate higher than in adults. While the small sample size and retrospective design limit the strength of the conclusions, these findings suggest that tolerability and patient preference are major challenges for risperidone LAI in early-onset schizophrenia. If longer-term prospective trials with larger samples confirm its efficacy and safety in this population, risperidone LAI could become an important treatment option for adolescents struggling with this severe disorder [26].

A case report from Turkey described an 11-year-old girl with early-onset bipolar disorder and severe non-adherence. She was switched to risperidone LAI 25 mg every two weeks, which led to rapid clinical improvement and return to school. However, treatment was stopped after two months due to marked weight gain and blurred vision. The case highlights both the potential benefits and the tolerability challenges of risperidone LAI in pediatric bipolar disorder [27].

## 3. Paliperidone Palmitate

Direct evidence on the use of paliperidone palmitate LAI in adolescents is still limited, but a series of clinical reports and observational studies is gradually emerging (Table 2). A 12-month retrospective study compared 18 adolescents with first-episode schizophrenia treated with paliperidone palmitate LAI (mean age 16.9 years, SD: 0.9) against peers maintained on oral risperidone. While both groups improved significantly, those on paliperidone palmitate LAI demonstrated greater reductions in PANSS and CGI-S scores, higher functional gains on Personal and Social Performance Scale (PSP), fewer hospitalizations, and higher satisfaction with treatment convenience. Considering side weight gain was more present in the oral risperidone group [28].

Two late-adolescent patients with severe psychotic disorders were stabilized after an initial loading regimen followed by monthly injections of paliperidone palmitate LAI, showing marked and sustained improvements in symptoms and functioning for at least one year; tolerability was good overall, although one patient experienced an acute oculogyric crisis after the first dose that resolved with anticholinergic treatment [29]. Real-world evidence is also provided by a cohort of 26 youths with autism spectrum disorder and/or intellectual disability who were switched to paliperidone palmitate LAI because of poor adherence and severe behavioral disturbance. Over a mean of 21.1 months (SD: 14.0; range 1–45), both psychiatric emergency visits and hospital admissions declined significantly (mean reduction 1.2 events; *p* = 0.02), and 62.5% of patients were rated as CGI-I responders at discharge. BMI rose slightly (mean +1.1 points), but no patient discontinued due to weight gain. Overall, 30.8% discontinued treatment, with half of these cases (15.4%) due to adverse events such as extrapyramidal reactions, oculogyric crisis, hyperprolactinemia, or autonomic symptoms post-injection, often at higher doses (156–234 mg every 3–4 weeks) [30]. Case reports further enrich the picture: a 16-year-old with schizophrenia and premenstrual exacerbations achieved stable remission on paliperidone palmitate LAI without notable side effects [31].

A small inpatient case series from the United States [32] described nine adolescents aged 14–17 years (seven males, two females) with severe psychiatric disorders who were started on long-acting injectables during hospitalization, primarily due to non-adherence. Diagnoses included schizophrenia (*n* = 5), schizoaffective disorder (*n* = 1), bipolar I disorder (*n* = 1), bipolar not otherwise specified (NOS) (*n* = 1), and mood disorder NOS (*n* = 1), with comorbidities such as substance use disorders, Post Traumatic Stress Disorder (PTSD), and ADHD being frequent. Five patients received paliperidone palmitate, while the remainder were treated with risperidone, aripiprazole, or fluphenazine LAIs. The mean hospital stay was 13.7 days (range 6–26). Clinical severity improved substantially, with both amelioration of CGI-S and CGI-I scores. Adverse effects were limited: some patients developed EPS (including neck stiffness and rigidity with risperidone and paliperidone) but these were managed successfully with benztropine.

## 4. Aripiprazole

Although direct evidence on long-acting aripiprazole in adolescents and children is still limited, converging data from aripiprazole once-monthly (AOM) and aripiprazole lauroxil (AL) are emerging from observational cohorts and case reports (Table 3). In a 30-patient inpatient cohort (mean age 16.3 ± 1.3 years), AOM was the most frequently prescribed second-generation LAI (40%); global functioning improved substantially from admission to discharge, short-term adverse events were less frequent with aripiprazole than with risperidone (*p* = 0.014), and LAIs were typically initiated for poor adherence (90%) and/or poor insight (73.3%). An inpatient case series initiating AL in 12 adolescents (mean age 16 ± 1; 58% female), almost always for nonadherence, reported acceptable short-term tolerability (one injection-site pain; one post-discharge discontinuation due to anxiety) and widely variable time-to-readmission among those rehospitalized (15–658 days), underscoring feasibility while highlighting the need for longer-term outcomes [33]. Case reports also describe the EMA-approved “two-injection start” for AOM (two 400 mg IM injections plus a single 20 mg oral dose on day 1), enabling rapid initiation without a 14-day oral overlap: a 16-year-old with schizophrenia improved from PANSS 136 to 43 and CGI 7 to 2 at one month with good tolerability and no akathisia [34], and a 16-year-old with bipolar I mania showed progressive improvement over four to five weeks (e.g., YMRS 34→4) with no akathisia or metabolic adverse effects [35]. Earlier U.S. inpatient experience (*n* = 9; ages 14–17) similarly used LAIs for nonadherence and included one adolescent started on AOM 400 mg who improved from CGI-S 7 to 1 by discharge [32]. Finally, outside typical inpatient settings, a justice-involved 15-year-old with schizophrenia remained stable and avoided re-incarceration over 12 months after transition to monthly LAI aripiprazole, illustrating adherence-related benefits in high-risk contexts [36].

## 5. Other Antipsychotics

Although the evidence is limited, there are reports of other long-acting injectable antipsychotics used in children and adolescents beyond paliperidone, risperidone, and aripiprazole. A 10-year retrospective audit from India identified 45 patients (mean age 14.7 years) treated with typical LAIs: fluphenazine decanoate (60.5%), flupenthixol decanoate (34.2%), and zuclopenthixol decanoate (18.4%). Baseline CGAS was 37.8 (SD: 5), and after an average follow-up of 17.8 months, 31 of 34 patients with complete data showed clinical improvement and functional gains. Still, adverse events were frequent: 47% experienced acute side effects, mostly tremor, rigidity, sialorrhea, and bradykinesia, with mean doses of ~31 mg for fluphenazine, 25 mg for flupenthixol, and 200 mg for zuclopenthixol [37]. Evidence from an adolescent forensic unit described three highly treatment-resistant and violent patients treated with olanzapine pamoate (up to 405 mg every 2 weeks) and zuclopenthixol decanoate (up to 600 mg every 2 weeks), sometimes in combination when clozapine was not an option. Over 12 months, BPRS scores fell from 89 to 44 (*p* = 0.005), and all three were successfully transferred to less restrictive settings; tolerability was acceptable, with only mild parkinsonism and bradykinesia [38]. Smaller series also mention the use of fluphenazine decanoate in U.S. inpatient units: in nine adolescents started on LAIs for non-adherence, overall CGI severity improved by discharge, with EPS linked to risperidone and paliperidone rather than to fluphenazine [32]. In a larger U.S. retrospective review of 45 pediatric inpatients initiated on LAIs, haloperidol decanoate accounted for 2.2% of cases, while most others received paliperidone or aripiprazole. Notably, 17 patients (37.8%) were prescribed off-label loading or maintenance regimens differing from adult labeling, and 6-month readmission occurred in 31.1% [39]. Finally, a systematic review of youth LAIs highlighted that most published reports were favorable but methodologically weak; analysis of 587 FDA MedWatch reports found EPS in 18%, neuroleptic malignant syndrome in 3%, and deaths in 2%, emphasizing the need for careful patient selection and close monitoring [40].

## 6. Discussion and Clinical Recommendations

### 6.1. Adolescent Vulnerability, Non-Adherence, and the Rationale for LAI Use

Adolescence is a brief but critical period of greatly increased susceptibility to psychiatric illness [41,42,43], and it is during these years that conditions such as schizophrenia, bipolar disorder, and schizoaffective disorder often first present with psychotic symptoms [2,44,45]. Recognition and treatment are complicated by the fact that adolescents may be reluctant to take medication [46]. At the same time, frequent changes in symptoms and the rapid evolution of their habits of thought and personality make it difficult to define any fixed therapeutic target [47]. Unsurprisingly, poor adherence to oral antipsychotics is common in this age group [46,48].

From a practical standpoint, LAI formulations can sometimes make treatment easier. In addition to ensuring steady levels of medication in the bloodstream, LAIs remove the burden of daily pill-taking and help avoid the peak–trough fluctuations of oral medication, which are often responsible for side effects such as sedation [12,19,49]. That said, no regulatory approvals or pediatric guidelines currently endorse LAI use in minors. The standard of care, therefore, remains to follow established recommendations and use oral antipsychotics with proven evidence in younger patients. In exceptional circumstances, however, LAIs are used off-label in clinical practice.

Because pediatric approvals and large randomized trials are lacking, guidance on adolescent LAI use largely comes from expert consensus and adaptations of adult recommendations. International documents on early-onset schizophrenia, such as the UK NICE guideline, do not recommend depots as first-line treatment because none are licensed for under-18s [50]. Instead, oral antipsychotics combined with psychosocial interventions remain the established standard [50]. At the same time, these guidelines acknowledge the high prevalence of non-adherence and the associated risk of relapse [50]. Clinical experience has therefore led many experts to consider LAIs for adolescents with severe, persistent psychotic disorders, or bipolar disorder with psychotic features, particularly when there is a history of poor adherence to oral therapy.

The present evidence suggests that the clinical benefits of LAIs—such as improved adherence, reduced relapse, and lower hospitalization rates—can be mechanistically explained by their pharmacokinetic and pharmacodynamic features, namely sustained plasma exposure, reduced peak–trough variability, and stable D_2_ receptor occupancy [10,11,12].

### 6.2. Considerations on LAIs and SUD in Adolescents

Across the reviewed studies, SUDs, most often involving cannabis, were present as comorbidities among adolescents initiated on LAIs, typically in inpatient or forensic settings [23,24,32]. Generally, these comorbidities complicate treatment by increasing non-adherence, reducing insight, and heightening the risk of relapse and rehospitalization [7,8,9]. In this context, LAIs may provide distinct advantages: by removing the need for daily dosing and limiting access to take-home pills, they can reduce the likelihood of misuse, diversion, or overdose, which is especially relevant in youths with dual diagnoses [7,8]. From a pharmacological perspective, the long half-life and stable plasma levels of LAIs may improve tolerability compared with oral formulations, potentially lowering discontinuation rates associated with adverse effects [12]. Nevertheless, important safety concerns remain. Off-label use in under-18s is still the norm rather than the exception, and limited evidence indicates that discontinuation due to adverse events may be more frequent in adolescents than in adults [26]. For this reason, clinical management should incorporate systematic SUD screening, early intervention strategies such as motivational interviewing, and close collaboration with addiction services to address substance use alongside psychiatric stabilization [41,50]. Psychoeducation for both patients and caregivers, close monitoring, and careful dose titration are also critical to balance the potential benefits of LAIs with the specific vulnerabilities of this high-risk group [51,52]. Ultimately, while preliminary evidence supports feasibility and possible benefit, well-designed prospective studies are needed to establish the role of LAIs in adolescents with dual diagnoses and to guide evidence-based recommendations.

### 6.3. Metabolic, Neurological, and Sociocultural Considerations

For adolescents, weight and height are often affected by the metabolic side effects of antipsychotic drugs, such as weight gain, hyperlipidemia, insulin resistance, and hyperprolactinemia [53,54]. Most studies report only basic metabolic outcomes in the short-term, and there are currently no data on the long-term cardiometabolic risks associated with these treatments. The risk of EPS, including dystonia, akathisia, and parkinsonism, also appears elevated in some reports involving paliperidone and risperidone, although results remain inconsistent across studies [22,26,30,32]. Sociocultural factors represent another key dimension influencing adherence and treatment outcomes. Active family involvement is often crucial to ensure continuity of care, especially in patients with limited decision-making capacity. Stigma toward injectable treatments can reduce acceptance among adolescents and their caregivers, particularly during the early stages of illness or in community-based settings [55]. Practical barriers such as limited access to specialized psychiatric services, transportation difficulties, and school or work commitments are rarely discussed in the literature. Nevertheless, these factors may be as critical as medical considerations in determining whether such treatments are feasible in real-world settings.

### 6.4. Clinical Recommendations

Whenever possible, like in adults, the initial step should be to start with the oral form of the antipsychotic, in order to establish both efficacy and tolerability, before switching to its LAI formulation [56]. Shared decision-making is crucial: obtaining the adolescent’s assent and the caregivers’ consent requires discussing the rationale for treatment, possible side effects, and the discomfort associated with injections. Although many young patients initially resist injections, acceptance is often good once therapy begins. For example, Benarous et al. emphasized acceptance in their review, and in some clinical cohorts of adolescents with bipolar disorder treated with LAIs, there were no dropouts—suggesting that once patients experienced the benefits firsthand, they were willing to continue [57].

A systematic review by Baeza et al., which pooled 13 studies on LAI use in children and adolescents, concluded that outcomes were generally positive, with clinical improvements reported in nearly every study. The safety profile was largely comparable to adults, reinforcing the idea that LAIs may be as effective in young patients as they are in older ones [58]. Given that no LAI is currently approved for individuals under 18 years of age, their use in adolescents constitutes off-label prescribing, although some studies considered this off-label use in youth [59]. Off-label use should be supported by a clear rationale, grounded in the best available evidence and expert consensus, and documented in the medical record. Informed consent from legal guardians is essential, as is the adolescent’s assent whenever appropriate, in line with international pediatric guidelines [60]. Clinicians must ensure that a thorough risk–benefit assessment is documented for each case, especially when alternative options are limited or adherence is compromised [60]. Informed consent from legal guardians is essential, along with the adolescent’s assent when developmentally appropriate [61]. Discussions should clearly outline the rationale for off-label use, potential risks (including metabolic and neurological side effects), and the lack of long-term data in pediatric populations. Risk management should include regular clinical monitoring and, when feasible, involvement of ethics committees or institutional oversight for complex cases [61].

In practical terms, LAIs should never be considered a first-line intervention in adolescents, but when clinical severity and adherence problems make their use necessary, several principles should guide practice:

(1) Stabilize on the oral form first, to ensure clinical response and tolerability before switching.
(2) Use the lowest effective dose possible, tailored to age and body size, and whenever possible, choose antipsychotics with the most favorable tolerability profile; starting doses should be conservative, following adult guidelines but adjusting for age and weight when needed. Dose escalation should proceed cautiously, with close clinical observation.
(3) Education and consent: provide detailed psychoeducation for both the patient and their family about what LAI treatment involves, balancing potential benefits (symptom control, easier adherence) against concerns (needle discomfort, clinic visits). Psychotherapy and ongoing psychosocial support should always be accompanied by pharmacological treatment.
(4) Monitor closely and systematically: ECG monitoring with calculation of QTc should be included to detect early cardiac risks. Routine follow-up should include body weight and body mass index (BMI) tracking, serum prolactin levels, assessment of extrapyramidal symptoms (EPS) using validated tools such as the Abnormal Involuntary Movement Scale (AIMS) or the Simpson–Angus Scale (SAS), fasting plasma glucose, and a full lipid profile.
(5) Frequent reassessment: adolescents on LAIs should be reviewed very regularly to evaluate whether improved adherence is translating into real-world benefits such as clinical stabilization, return to school, or fewer hospitalizations. If not, the treatment plan should be reconsidered.
(6) Integration with psychosocial care: Family psychoeducation is critical, both to promote adherence and to address stigma or resistance to injectable medications. A multidisciplinary team—ideally involving child psychiatrists, case managers, and psychologists—should accompany pharmacologic interventions. School coordination and peer support may also be important in promoting long-term functioning and treatment engagement.

Adolescents with co-occurring substance use disorders (SUD) should also be managed with specific considerations:

(1) Systematic screening for SUD should be conducted at baseline and follow-up. Brief interventions are recommended, with motivational interviewing as the first-line approach, and referrals to addiction services should be arranged when indicated.
(2) Adherence support for individuals with dual diagnoses requires reminders, family involvement, and effective case management. LAIs are particularly valuable when non-adherence to oral medication is driven by SUD.
(3) Mitigating misuse and diversion risks is another priority: LAIs are generally preferred when the availability of take-home pills could lead to intentional misuse or diversion. Missed injections should be documented promptly, and barriers such as transport, clinic access, or stigma need to be addressed without delay.
(4) Off-label use and risk–benefit evaluation must be considered: since LAIs are not licensed for individuals under 18 years, individualized risk–benefit discussions should be documented, caregiver consent obtained, and adolescent assent secured. Close vigilance for adverse effects (including EPS, prolactin-related changes, and anxiety/activation) is required, with a low threshold for dose adjustment or switching.

### 6.5. Limitations and Future Perspectives

This review is subject to several limitations, which mostly reflect the current state of the literature. Most available studies are small and preliminary, often retrospective, and frequently based on case series without control groups. To date, no RCTs have been conducted on LAIs in adolescents. This leaves the evidence base weak, calling for caution when drawing conclusions. The included studies vary widely in sample size, diagnostic categories, treatment settings (inpatient vs. outpatient), and dosing regimens. Such heterogeneity makes it difficult to compare findings across studies or to extract consistent clinical guidance. Key outcomes—such as functional recovery, relapse rates, or quality of life—are often inconsistently reported and rarely assessed over time. In most cases, follow-up periods are brief, which limits our understanding of long-term safety, effectiveness, and adherence. Moreover, the lack of demographic diversity in most samples further restricts the generalizability of the findings. Finally, important areas such as cardiometabolic risks, neurological safety, and sociocultural determinants of treatment adherence are seldom examined systematically. Altogether, these gaps underscore the need for well-designed, prospective, large-scale trials that utilize standardized outcome measures. The off-label use of LAIs in adolescents represents a relevant case study for the intersection between drug formulation science, clinical pharmacology, and real-world psychiatric care. Future research should aim to characterize age-specific pharmacokinetics, dosing strategies, and long-term safety in this population.

## Figures and Tables

**Table 1:** Summary of Studies on Risperidone Long-Acting Injection in Adolescents.

Study	Sample Size (*n*)	Mean Age (SD)	Diagnosis	Dose	Duration	Main Outcomes	Adverse Events
Boarati et al. (2013) [21]	19	12.1 (2.2)	Early-onset bipolar disorder	25–37.5 mg q2w	6 months	CGI-S, CGAS	No severe AE; no significant metabolic changes
Ardic et al. (2018) [22]	11	14.9 (1.1)	Conduct disorder and aggressive behaviours	25 mg q2w	Retrospective, inpatient (not specified)	CGI-S, CGI-I	Mild EPS
Demirkaya et al. (2017) [23]	14	13.9 (2.9)	Conduct disorder	25 mg q2w	Retrospective, inpatient (short-term)	CGI-S, CGI-I	Mild Sedation, weight gain, rigidity; no severe AEs
Fortea et al. (2018) [24]	30 (36.7% treated with risperidone)	16.3 (1.3)	Mixed psychiatric diagnoses (psychotic disorders, disruptive behavior disorders)	Not specified	Length of hospitalization (not specified)	CGAS	More short-term AEs with risperidone vs. aripiprazole. No severe AEs
Ruan et al. (2010) [25]	31	15.9 (3.3)	Early-onset schizophrenia	25–50 mg q2w	24 weeks	PANSS, CGI-S, CGI-I	Mild to moderate AE, one patient discontinued treatment
Suzuki et al. (2017) [26]	15 (compared with 48 adults)	16.8 (1.7)	Early-onset schizophrenia	25–50 mg q2w	1 year	CGI-S, CGI-I, BPRS, DIEPSS	Higher discontinuation due to side effects in adolescents; common AEs included EPS and metabolic issues
Karakoc et al. (Case report) [27]	1	16	Early-onset bipolar disorder	25 mg q2w	2 months	YMRS	Significant weight gain; blurred vision, which lead to discontinuation

BPRS: Brief Psychiatric Rating Scale; CGAS: Children’s Global Assessment Scale; CGI-I: Clinical Global Impressions–Improvement; CGI-S: Clinical Global Impressions–Severity; DIEPSS: Drug-Induced Extrapyramidal Symptoms Scale; PANSS: Positive and Negative Syndrome Scale; YMRS: Young Mania Rating Scale.

**Table 2:** Summary of Studies on Paliperidone Long-Acting Injection in Adolescents.

Study	Sample Size (*n*)	Mean Age (SD)	Diagnosis	Dose	Duration	Main Outcomes	Adverse Events
Petrić et al. (2019) [28]	36 (18 PPLAI; 18 oral risperidone)	NR (Age range: 15–18)	First-episode schizophrenia (adolescents)	Common loading 150 mg (72.2%) or 100 mg (27.8%); median maintenance 75 mg at 12 months	12 months	PANSS, CGI-I, CGI-S, PSP, TSQM	PPLAI: 5.5% hyperprolactinemia, 5.5% weight gain; Oral: 5.5% hyperprolactinemia, 16.7% weight gain.
Fàbrega et al. (2015) [29]	2	14 (patient 1); 17 (patient 2)	Undifferentiated schizophrenia (patient 1); Psychotic disorder NOS and Conduct Disorder (patient 2)	PPLAI monthly (50 mg; with an early repeat after 1 week)	Up to 12 months (patient 1); 1 month (patient 2)	PANSS, GAF, UKU side effects rating scale (patient 1)	None (patient 1), Oculogyric crisis, somnolence (patient 2)
Fortea et al. (2018) [24]	30 (23.3% treated with paliperidone	16.3 (1.3)	Mixed psychiatric diagnoses (psychotic disorders, disruptive behavior disorders)	NR	Length of hospitalization (not specified)	CGAS	More short-term AEs with risperidone vs. aripiprazole. No severe AEs
Simpson et al. (2024) [30]	26	NR (Range: 3–20 years)	Autism/intellectual disability	Typical maintenance 156 mg q4wk (range included q3wk or up to 234 mg)	Variable; mean time on PPLAI ≈ 21 months (service cohort)	CGI-I, CGI-S, Hospital Presentations	Discontinuations related to EPS (incl. oculogyric crisis), hyperprolactinemia; mild BMI change
Wang et al. (2023) [31]	1	16 years	Schizophrenia with premenstrual exacerbations	150 mg loading, then 100 mg monthly	Up to 3 years	PANSS	No serious AEs; no clinically significant lab/ECG changes reported.
Pope & Zaraa (2016) [32]	9 (Paliperidone LAI: 5)	NR (Range: 14–17)	|Schizophrenia (*n* = 5), schizoaffective (*n* = 1), bipolar I (*n* = 1), bipolar NOS (*n* = 1), mood disorder NOS (*n* = 1)	NR	NR	CGI-I, CGI-S	EPS in some cases (neck stiffness/rigidity), managed with benztropine; otherwise well tolerated

CGAS: Children’s Global Assessment Scale; CGI-I: Clinical Global Impressions–Improvement; CGI-S: Clinical Global Impressions–Severity; GAF: Global Assessment of Functioning; PANSS: Positive and Negative Syndrome Scale; PSP: Personal and Social Performance Scale; TSQM: Treatment Satisfaction Questionnaire for Medication; UKU: Udvalg for Kliniske Undersøgelser Side Effect Rating Scale.

**Table 3:** Summary of Studies on Aripiprazole Long-Acting Injection in Adolescents.

Study	Sample Size (*n*)	Mean Age (SD)	Diagnosis	Dose	Duration	Main Outcomes	Adverse Events
Fortea et al. (2018) [24]	30 (40% treated with aripiprazole	16.3 (1.3)	Mixed psychiatric diagnoses (psychotic disorders, disruptive behavior disorders)	Not specified	Length of hospitalization (not specified)	CGAS	More short-term AEs with risperidone vs. aripiprazole. No severe AEs
Salvi et al. (2022) [34]	1	16	Schizophrenia	“two-injection start”: 400 mg × 2 IM + 20 mg oral on Day 1	1 month follow-up	PANSS, CGI 7	None reported; no akathisia
Orsolini et al. (2024) [35]	1	16	Bipolar I mania (with SUD)	“two-injection start” 400 mg × 2 IM + 20 mg oral on Day 1	5 weeks	YMRS, PANSS, CGI	None reported; no akathisia
Moon et al. (2023) [33]	12	16 ± 1	Bipolar Disorder (25%), schizophrenia (17%), Major Depressive Disorder with psychosis (17%), mood disorder NOS (17%), others	NR	Initiated during hospitalization; among those rehospitalized, time-to-readmission 15–658 days	Readmission, Tolerability	1 injection-site pain; 1 post-discharge discontinuation due to anxiety
Pope & Zaraa (2016) [32]	9 (11% treated with aripiprazole)	14–17 (mean NR)	Schizophrenia (*n* = 5), schizoaffective (*n* = 1), bipolar I (*n* = 1), bipolar NOS (*n* = 1), mood NOS (*n* = 1)	AOM 400 mg (single patient)	NR	CGI-I, CGI-S	None

AOM: aripiprazole once-monthly, CGAS: Children’s Global Assessment Scale; CGI-I: Clinical Global Impressions–Improvement; CGI-S: Clinical Global Impressions–Severity; PANSS: Positive and Negative Syndrome Scale; YMRS: Young Mania Rating Scale.

## Data Availability

No new data were created or analyzed in this study.

## Abbreviations

The following abbreviations are used in this manuscript:

ADHD	Attention-Deficit/Hyperactivity Disorder
AE/AEs	Adverse Event(s)
AL	Aripiprazole Lauroxil
AOM	Aripiprazole Once-Monthly
ASD	Autism Spectrum Disorder
BPRS	Brief Psychiatric Rating Scale
BMI	Body Mass Index
CGI-I	Clinical Global Impressions—Improvement
CGI-S	Clinical Global Impressions—Severity
CGAS	Children’s Global Assessment Scale
DIEPSS	Drug-Induced Extrapyramidal Symptoms Scale
ECG	Electrocardiogram
EMA	European Medicines Agency
EPS	Extrapyramidal Symptoms
FDA	U.S. Food and Drug Administration
GAF	Global Assessment of Functioning
ID	Intellectual Disability
IM	Intramuscular
LAI/LAIs	Long-Acting Injectable (antipsychotic [s])
MDD	Major Depressive Disorder
NICE	National Institute for Health and Care Excellence
NOS	Not Otherwise Specified
NR	Not Reported
PANSS	Positive and Negative Syndrome Scale
PSP	Personal and Social Performance (Scale)
PTSD	Post-Traumatic Stress Disorder
q2w	Every 2 weeks
q3wk	Every 3 weeks
q4wk	Every 4 weeks
RCT/RCTs	Randomized Controlled Trial(s)
SUD	Substance Use Disorder
TSQM	Treatment Satisfaction Questionnaire for Medication
UKU	Udvalg for Kliniske Undersøgelser Side Effect Rating Scale
YMRS	Young Mania Rating Scale
